# Skeletal Muscle Mitochondrial Energetic Efficiency and Aging

**DOI:** 10.3390/ijms160510674

**Published:** 2015-05-11

**Authors:** Raffaella Crescenzo, Francesca Bianco, Arianna Mazzoli, Antonia Giacco, Giovanna Liverini, Susanna Iossa

**Affiliations:** Department of Biology, University of Naples “Federico II”, Napoli I-80126, Italy; E-Mails: rcrescen@unina.it (R.C.); francibianco@yahoo.it (F.B.); arimazzoli@hotmail.it (A.M.); antonia.giacco@hotmail.it (A.G.); liverini@unina.it (G.L.)

**Keywords:** mitochondria, aging, skeletal muscle, proton leak

## Abstract

Aging is associated with a progressive loss of maximal cell functionality, and mitochondria are considered a key factor in aging process, since they determine the ATP availability in the cells. Mitochondrial performance during aging in skeletal muscle is reported to be either decreased or unchanged. This heterogeneity of results could partly be due to the method used to assess mitochondrial performance. In addition, in skeletal muscle the mitochondrial population is heterogeneous, composed of subsarcolemmal and intermyofibrillar mitochondria. Therefore, the purpose of the present review is to summarize the results obtained on the functionality of the above mitochondrial populations during aging, taking into account that the mitochondrial performance depends on organelle number, organelle activity, and energetic efficiency of the mitochondrial machinery in synthesizing ATP from the oxidation of fuels.

## 1. Introduction

Increasing age leads to a decline in cell functionality, generally termed as “aging” [1]. All tissue and organs of the body are involved in the phenomenon of aging, but the extent of the cellular impairment is greatly variable, since post-mitotic tissues are the most sensitive targets of the aging process [2]. Among the latter, skeletal muscle tissue is profoundly affected during aging, and its functional decline is characterized by a progressive atrophy, that becomes more severe with advancing age and that from a certain point onwards can lead to mobility impairment, increased risk of falls, and physical frailty [3,4]. The loss of function in skeletal muscle is also responsible for the development of age-associated insulin resistance and related metabolic disturbances [5,6,7,8,9]. For these reasons, understanding the mechanisms underlying aging in skeletal muscle is fundamental for promotion of health and mobility in the elderly. To this end, the most used animal model of human aging is represented by ad libitum fed caged rodents, that exhibit a sedentary lifestyle and unrestricted diet, and that develop an age-induced spontaneous obesity and insulin resistance already at 4 months of age [7].

Although the loss of skeletal muscle protein mass during aging could at least partially explain the decline in muscle performance, a role for mitochondria has been postulated in the aging process [10,11]. In fact, mitochondria have a major role in energetic homeostasis by determining ATP availability in the cells. A decrease in mitochondrial function could therefore cause an inability to meet cellular ATP demands, so that skeletal muscle cells lose their capacity to adapt to physiological stress imposed across the entire lifespan [12]. In addition, dysfunctional mitochondria could contribute to the development of age-induced insulin resistance [13], since mitochondrial oxidative capacity has been considered a good predictor of insulin sensitivity [14].

**Figure 1:**
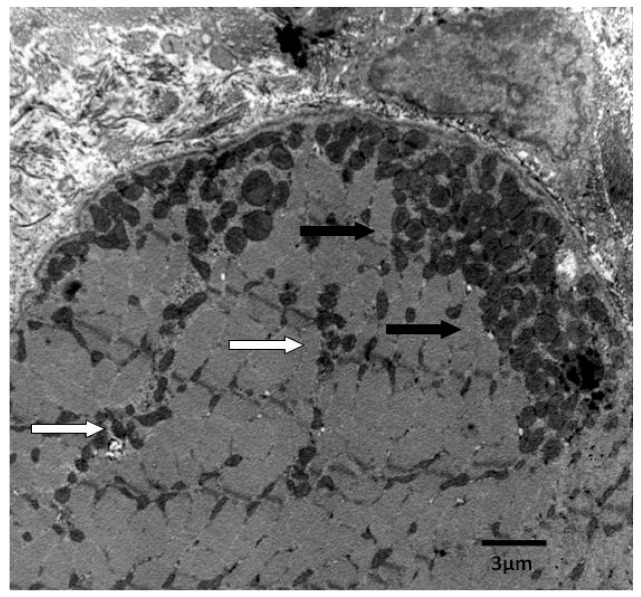
Heterogeneous mitochondrial populations in skeletal muscle cells. Scale bar = 3 µm. White arrows = Intermyofibrillar mitochondria; Black arrows = Subsarcolemmal mitochondria.

One important issue that should be taken into account when studying mitochondria in skeletal muscle is that the mitochondrial population is heterogeneous, composed of mitochondria located either beneath the sarcolemmal membrane (subsarcolemmal, SS) or between the myofibrils (intermyofibrillar, IMF) [15] (Figure 1). Since these two mitochondrial populations exhibit different energetic characteristics and can be differently affected by physiological stimuli [16], it is important that both are separately studied. Therefore, the purpose of the present review is to summarize the results obtained on the functionality of the above mitochondrial populations during aging, taking into account that the mitochondrial performance depends on organelle number, organelle activity, and energetic efficiency of the mitochondrial machinery in synthesizing ATP from the oxidation of fuels. A search in PubMed of relevant articles was conducted, by using query “skeletal muscle mitochondria and aging”, “subsarcolemmal mitochondria and aging”, and “intermyofibrillar mitochondria and aging”, with the inclusion of related articles by the same groups.

## 2. Age-Related Changes in Mitochondria

Although many studies have addressed the issue of mitochondrial performance attenuation during aging in skeletal muscle, the obtained results are contradictory, with some papers reporting impairment of mitochondria with increasing age [17,18,19,20,21,22,23] and others showing no age-induced change [24,25], underscoring the complexity of understanding in this area.

The discrepancy between the published data can partly be explained by differences in experimental approach. However, even when comparing the results obtained with similar methodological approach, divergent outcomes are evident. For example, among the studies that have measured the activity of enzymatic complexes, such as citrate synthase and electron transport chain complexes, to obtain indirect insights into the energy producing (respiratory) capacity of the mitochondria, some of them have reported an age-dependent decrease in aging muscle [26,27,28] while other studies found no variation [29] or reported a variable response in different muscles [30]. An alternative approach used to study mitochondrial alteration in aging muscle is assessing total mitochondrial content. Again, some studies reported that mitochondrial content is reduced in aging muscle [31,32] and others found no change [21,33,34,35]. There is however consensus on the finding that aging skeletal muscle has a blunted capacity for generation of new mitochondria in response to both endurance exercise training [36] and chronic electrical stimulation [37,38].

To our knowledge, studies carried out specifically on mitochondria located beneath the sarcolemmal membrane (SS) and between the myofibrils (IMF) from skeletal muscle during aging are scarce. In a pioneering research, Farrar *et al.* [39] found that ADP-stimulated respiration did not change with increasing age in IMF and SS mitochondria, while aging decreased the amount of IMF proteins. Chabi *et al.* [32] studied senescent (3 years) rats and have found that in SS and IMF mitochondria the capacity for ATP production was reduced, as a result of diminished mitochondrial content per gram of muscle. Drew *et al.* [40] found a decrease in ATP production between 12 and 26 months of age in SS mitochondria from gastrocnemius muscle in Fisher rats. Very few studies specifically addressed the differential regulation of SS and IMF mitochondria with aging in humans. These studies evaluated the SS and IMF mitochondrial content of skeletal muscle in young and old men and reported no age-dependent change in both mitochondrial populations [34,41]. From all the above results, it emerges a differential effect of aging on SS and IMF mitochondria, at least in animal models. More studies on humans are needed to validate the differential effect of aging on the two mitochondrial populations. In addition, studies on changes in mitochondrial function induced by aging or other physiological stimuli should be carried out on the two different mitochondrial populations existing in skeletal muscle.

## 3. Mitochondrial Energetic Efficiency during Aging

**Figure 2:**
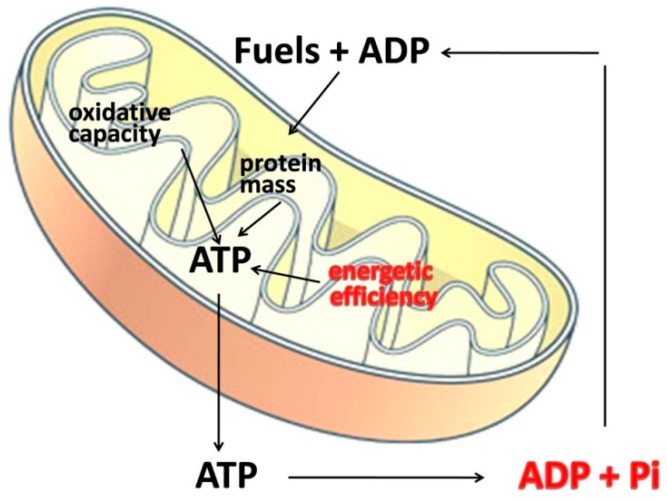
Factors affecting cellular fuel oxidation. The amount of burned fuels mainly depends on mitochondrial energetic efficiency and ATP turnover (in red). Mitochondrial mass and oxidative capacity are less important because mitochondria are thought to have a much greater capacity to generate ATP than what is usually required [42].

From the analysis of the published results on the differential effect of aging on IMF and SS mitochondria, no clear picture can be obtained, partly because of differences in the experimental approach used to evaluate mitochondrial function. The mitochondrial performance depends on organelle number, organelle activity, and energetic efficiency of the mitochondrial machinery in synthesizing ATP from the oxidation of fuels. In addition, it is well known that the amount of fuels oxidized by the cell is dictated mainly by ATP turnover rather than by mitochondrial oxidative capacity and therefore a decrease in mitochondrial capacity and/or number becomes more important when cells increase their metabolic activity, *i.e.*, during contraction, while an increased mitochondrial efficiency still alter the amount of oxidized fuels also at rest (Figure 2). The efficiency with which dietary calories are converted to ATP is determined by the degree of coupling of oxidative phosphorylation. If the respiratory chain is highly efficient at pumping protons out of the mitochondrial inner membrane, and the ATP synthesis is highly efficient at converting the proton flow through its proton channel into ATP (from ADP), then the mitochondria will generate maximum ATP and minimum heat per calorie. In contrast, if the efficiency of proton pumping is reduced and/or more protons are required to make each ATP molecule, then each calorie will yield less ATP.

The main point of regulation of the oxidative phosphorylation efficiency [43] is represented by the degree of coupling between oxygen consumption and ATP synthesis, which is always lower than 1 and can vary according to the metabolic needs of the cell [44]. Among the factors, which affect mitochondrial degree of coupling, an important role is played by the permeability of the mitochondrial inner membrane to H^+^ ions (leak). It is now well known that mitochondrial inner membrane exhibit a basal proton leak pathway, whose contribution to the basal metabolic rate in rats has been estimated to be about 20%–25% [45]. In addition, it is well known that fatty acids can act as natural uncouplers of oxidative phosphorylation, by generating a fatty acid-dependent proton leak pathway [46,47,48], which is a function of the amount of unbound fatty acids in the cell.

The issue of mitochondrial efficiency has been explored in humans *in vivo*, and it has been found that the effect of aging is fiber type-dependent. In fact, in mildly uncoupled fiber types (*i.e.*, tibialis anterior) no age effect is evident, while a substantial uncoupling takes place with aging in well coupled fiber types (*i.e.*, dorsal interosseous) [49]. Due to the *in vivo* conditions, these studies do not distinguish between SS and IMF mitochondria. Since aging has been show to selectively affect IMF but not SS mitochondria in heart [50], and taking into account the above considerations, a study was carried out on the putative changes in mitochondrial performance and efficiency during aging in SS and IMF mitochondria. In the transition from young adulthood (60 days) to adulthood (180 days), SS and IMF skeletal muscle mitochondria displayed an increase in the degree of coupling and efficiency, as well as a decreased fatty acid dependent proton leak [51]. The above modifications in mitochondrial performance occurred concomitantly with an increase in whole body lipids and plasma non-esterified fatty acids (NEFA) [51], suggesting a link between skeletal muscle increased mitochondrial efficiency and metabolic impairments.

These results were extended to the evaluation of how the progression of aging affects skeletal muscle mitochondrial function by measuring mitochondrial respiratory capacity and proton leak in SS and IMF mitochondria from adult (six months) and old (two years) rats [52]. A significant decrease in oxidative capacity was found in skeletal muscle homogenates, as well as in SS and IMF mitochondria from old rats. Oxidative capacity measured in the homogenate reflects the product of mitochondrial mass and activity, while oxidative capacity in isolated organelles depends only on mitochondrial activity, and therefore the similar age-induced decrease in oxidative capacity in homogenates and isolated mitochondria found in old rats could be indicative of a lack of changes in mitochondrial mass. A decreased mitochondrial mass has been found in senescent rats (36 months old) [32], thus suggesting that an impairment of mitochondrial biogenesis occurs in late aging and/or it takes place selectively in specific muscles, such as gastrocnemius, whose mitochondrial mass has been found decreased in old rats [20,53].

Both mitochondrial populations from old rats exhibited a significant decrease in proton leak [52], suggesting that with increasing age the efficiency of oxidative phosphorylation increases both in SS and IMF mitochondria. Similar results have been obtained *in vivo* in aged rat skeletal muscle, where a trend for a higher coupling efficiency was found [20]. Skeletal muscle cells in sedentary laboratory rats operate in conditions of low ADP availability, near to state 4 (when no ADP is available), with a high contribution of proton leak to total oxygen consumed by mitochondria [54], and therefore the decreased proton leak found in SS and IMF mitochondria from old rats is physiologically relevant. When mitochondria are more efficient, less substrates are oxidized to obtain ATP. Therefore, the increased mitochondrial coupling in skeletal muscle could contribute to the decreased energy expenditure that is evident even after the decrease in lean mass has been taken into account and that is at the basis of age-induced obesity, since skeletal muscle energy metabolism accounts for about 30% of whole body energy expenditure in resting conditions [45].

One interesting question is: What could be the impact of the increased mitochondrial coupling on ATP yield? Although it is very difficult to calculate the theoretical difference in ATP yield per calorie in highly efficient *vs.* inefficient proton pumping, a rough estimate could be obtained using a published estimate of the control value of proton leak on *P*/*O* ratio in skeletal muscle [55]. This value is reported to be −0.72 [55], so we can calculate that the 40% decrease in proton leak found in old rats [52] would result in a 29% increase in *P*/*O* ratio, and therefore in the amount of ATP obtained per unit of oxygen consumed. However, when mitochondria are more coupled, ATP is produced at a slower rate [44,56], that could be unable to meet cellular energy demands, especially during skeletal muscle contraction. In fact, in elderly subjects it has been found that a lower speed of ATP production is associated with higher fatigability [57].

**Figure 3:**
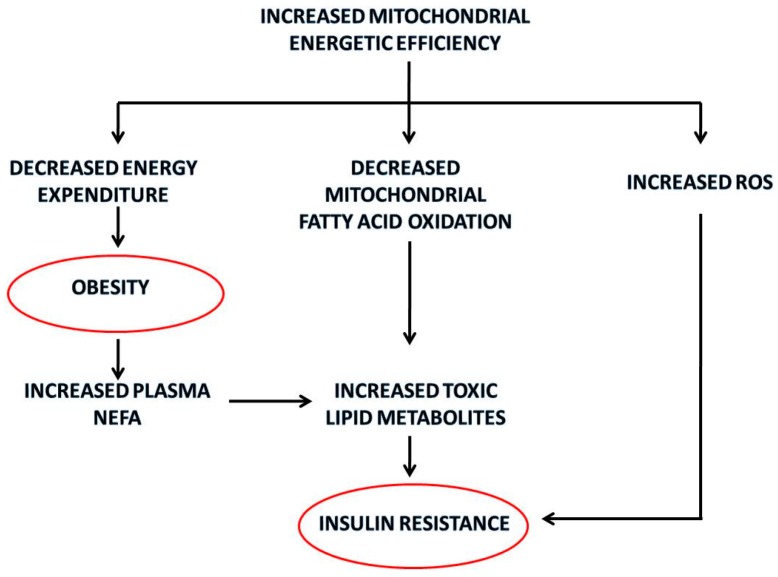
Summary of the metabolic implications of the increased mitochondrial energetic efficiency in skeletal muscle with aging. The red circles identify the pathological outcomes.

Another deleterious consequence of reduced substrate burning in skeletal muscle could be intracellular triglyceride accumulation and lipotoxicity, since NEFA serum levels are significantly higher in older rats. In fact, in conditions of increased plasma NEFA, more fatty acids enter in the cells and if they are poorly oxidized, they accumulate intracellularly in myocytes mainly as long-chain fatty acyl-CoA, monoacylglcyerol, diacylglycerol, phosphatidic acid, triacylglycerol and ceramide. Among these fatty acid derivatives, high intramyocellular levels of diacylglycerol and ceramides are directly associated with insulin resistance [58]. The metabolic implications of increased mitochondrial energetic efficiency are summarized in Figure 3.

## 4. Oxidative Stress in Aging Mitochondria

Reactive oxygen species (ROS) production increases when mitochondrial potential is higher [59,60], and therefore increased mitochondrial energetic efficiency could induce a condition of increased oxidative stress. In fact, one of the postulated physiological roles for the uncoupling effect of fatty acids is to maintain mitochondrial membrane potential below the critical threshold for ROS production, especially *in situ*ations of low rates of ATP turnover, such as in resting skeletal muscle [61]. It has been proposed that ROS generation during the normal oxidative activity of mitochondria leads to damage of lipids, proteins and DNA, especially in postmitotic tissues, such as skeletal muscle, and that this oxidative damage is at the basis of the biological phenomenon of cellular aging [10,11]. Therefore, the increased coupling of mitochondrial oxidative phosphorylation found in 180 day-old rats [51] could led to the oxidative damage of skeletal muscle cell, detectable later in life. However, the increased uncoupling protein 3 (UCP3) protein content found in SS and IMF mitochondria from 180 days old rats could be involved in the protection from oxidative damage [62,63]. In fact, it has been proposed that UCP3 translocates fatty acid peroxides from the inner to the outer membrane leaflet, thus preserving macromolecules from being oxidized by very aggressive fatty acid peroxides [62,63], while its uncoupling effect is considered very low, due to its low content in skeletal muscle mitochondria [64]. Therefore, the up-regulation of UCP3 during aging could buffer oxidative damage, that otherwise could be even higher.

In 2 year-old rats, an increase in the degree of lipid peroxidation was found only in SS mitochondria, although the decrease in proton leak is the same in both mitochondrial populations [52], while Chabi *et al.* [32] studied senescent (3 years) rats and found that ROS production was enhanced in SS and IMF mitochondria. The differential susceptibility to oxidative stress of SS and IMF mitochondria could depend on their different content of UCP3. In fact, a significant increase in UCP3 content was evident in SS and IMF mitochondria from old rats, but it was more marked in IMF mitochondria (about 10-fold) than in SS mitochondria (about 5-fold). Therefore, the lower increase in UCP3 content in SS mitochondria is probably the cause of the higher oxidative damage found in this mitochondrial population, while IMF mitochondria seem more protected by oxidative damage by the marked up-regulation of UCP3, so preserving the capacity to produce ATP for muscle contraction. One could speculate that, as aging proceeds, SS mitochondria undergo progressive oxidative damage with loss of functional activity. In fact, in senescent (30–36 month-old) animals, an increase in mitochondrial proton leak [65], a decrease in mitochondrial coupling [66] or a decrease in mitochondrial membrane potential in SS but not in IMF mitochondria has been found [32]. In conclusion, the increased coupling of SS mitochondria causes an increase of the oxidative damage in this mitochondrial population, that is located beneath the plasma membrane and provides ATP for membrane transports and signal transduction pathways [67]. On the other hand, IMF mitochondria, that provide ATP for muscle contraction, seems to be more protected from oxidative damage, and could thus increase their oxidative capacity and density in response to endurance training even in old age [68].

A decreased UCP3 content has been found in skeletal muscle mitochondria from old rats [31,69,70], but these results have been obtained using Fischer 344 rats, a rat strain that gain weight only moderately with age compared with other strains (*i.e.*, Sprague-Dawley, Wistar, Long Evans) [8]. Therefore, it can be hypothesized that the regulation of UCP3 with aging in skeletal muscle mitochondria is strain-dependent. Therefore, it is possible that age-induced oxidative damage in skeletal muscle and age-induced obesity are intimately linked. Further studies on the degree of obesity and oxidative damage induced by aging in different strains and species are needed to substantiate the hypothesis.

## 5. Conclusions

In the rat model of human obesity the progression of aging is accompanied by an increased efficiency of SS and IMF mitochondria but an increased oxidative damage occurs only in the SS population. Therefore, a differential susceptibility of SS and IMF mitochondria to aging-induced damage emerges, although more studies on humans are needed to validate the differential effect of aging on the two mitochondrial populations. These observations also indicate that studies on changes in mitochondrial function induced by aging or other physiological stimuli should be carried out on the two different mitochondrial populations existing in skeletal muscle.
